# Evaluation of contrast sensitivity and color vision in lead and zinc mine workers

**Published:** 2020

**Authors:** Farzaneh Fattahi, Mehdi Khabazkhoob, Ebrahim Jafarzadehpour, Ali Mirzajani, AbbasAli Yekta

**Affiliations:** * Noor Research Center for Ophthalmic Epidemiology, Noor Eye Hospital, Tehran, Iran; ** Department of Medical Surgical Nursing, School of Nursing and Midwifery, Shahid Beheshti University of Medical Sciences, Tehran, Iran; *** Department of Optometry, Faculty of Rehabilitation, Iran University of Medical Sciences, Tehran, Iran; **** Noor Ophthalmology Research Center, Noor Eye Hospital, Tehran, Iran; ***** Department of Optometry, School of Paramedical Sciences, Mashhad University of Medical Sciences, Mashhad, Iran

**Keywords:** contrast sensitivity, color vision, lead and zinc miners

## Abstract

**Purpose.** This study was performed to determine achromatic contrast sensitivity and color vision in lead and zinc mine workers.

**Methods.** A total of 230 male workers, who had been working in mine and had been in contact with minerals for at least 1 year, were considered as the case group, and the age of 90 years matched men who have not been in contact with minerals, being regarded as the control group. Contrast sensitivity was assessed using the Freiburg test at three frequencies of 1, 5 and 15 cycles of degree and under low mesopic light condition by two gratings and Landolt C stimuli. Color vision was assessed using the Farnsworth D-15 test under high mesopic light condition. Both tests were carried out monocularly. Data were analyzed using version 22 SPSS software.

**Results.** There was a significant difference between studied groups with Landolt C stimulus in all three frequencies 1, 5 and 15 cycles per degree (p=0.009, p=0.016 and p=0.003). With Grating stimulus, there was a significant difference between the two groups in frequencies of 1 and 15 cycles per degree but at frequency of 5 cycles per degree, there was a border difference (p<0.0001, p=0.051 and p=0.008). A significant difference was observed between color confusion indexes of the two groups (p<0.0001).

**Conclusion.** Chronic exposure to mineral in lead and zinc mine may cause color vision deficiency and decrease in contrast sensitivity. It is recommended that Farnsworth D-15 and Freiburg contrast sensitivity tests would be involved in the early diagnosis of neurodegenerative and visual disorders in workers exposed to minerals.

## Introduction

The effects of neurotoxic substances on contrast sensitivity have already been examined in many studies of the visual system. Contrast sensitivity measurements, have recently been used in the assessment of neurologic disorders caused by exposure to neurotoxic substances. Reduced contrast sensitivity among workers who were exposed to different neurotoxic substances have been reported. Toxic occupational exposure may affect the neuro-optic pathways [**1**].

The measurement of contrast sensitivity at grating various frequencies is a valuable tool for understanding visual mechanisms, which is incrementally used for studying visual and neural disorders [**2**].

In addition to contrast sensitivity, occupational exposure to neurotoxic substances may also have an impact on color vision. Perception of color is one of the sensory capabilities of the vision system. Color vision impairment is relatively common in the general population. It causes negative effects on a person’s daily and professional activities. Color vision impairment may be congenital (often red-green spectrum) or acquired. The essential causes of acquired color vision impairment include neurological disorders, visual disturbances, intoxication with neurotoxic drugs and occupational exposure to neurotoxic substances at workplace. However, it should be noted that other causes may also lead to the acquired color vision impairment, the most important ones being: aging, smoking, alcohol consumption, cerebrovascular disease, systemic disease with neurological complications (such as diabetes mellitus) and severe trauma to the head [**3**].

Loss of contrast sensitivity and impaired color vision following occupational exposure to different substances such as stern, organic solvents, heavy metals like mercury, lead, manganese, etc., have been reported [**4**-**9**].

Variation contrast sensitivity and color vision after exposure to industrial materials, solvents and heavy metals have been investigated in many studies, but these changes after exposure to minerals are rarely being investigated. Because mine environment has a different mineral composition with various structures that are features, also various gases may be emitted during work, probably because of exercise that is performed during excavations and purification of minerals. The aim of this study was to measure contrast sensitivity and color vision after exposure to minerals in Koushk lead and zinc mine.

## Methods

This cross-sectional comparative study was conducted at the Koushk mine. The exclusion criteria were the following: eye diseases such as cataract, glaucoma, corneal diseases, systemic diseases including hypertension, diabetes, neurological disorders including Alzheimer’s, Parkinson’s, multiple sclerosis and stroke, congenital color vision impairment, neurotoxin drugs use, such as chloroquine, digital and phenytoin, age over 50 years, corrected visual acuity with Snellen chart lower than 20/ 25 in any of the eyes and work experience lower than 1 year. After signing an informed consent, preliminary information (age, working sector, history of systemic and ocular diseases and job experience) were completed. At first, visual acuity was measured by Snellen chart at a distance of 4 meters and refractive error was determined by CHAROPS auto kerato-refractometer and Heine Beta 200 retinoscope and intraocular status was checked by Heine k180 ophthalmoscope. Then, if needed, optical correction with glasses and without glasses, in emmetropic cases, for each eye separately, D15-Fransworth color vision test Company Bernell and Freiburg contrast sensitivity test version 3.9.3, were performed.

Contrast sensitivity test: Freiburg contrast sensitivity test is a psychophysical test that has the capability to be downloaded from the Internet and be run on PC easily and at a low cost [**10**].

According to studies, the Freiburg test is presented as a valid, sensitive and reliable method for contrast sensitivity determination at different levels of brightness, with and without glare. The test shows a series of stimuli using best parameter estimation by sequential testing (PEST) algorithm and through responses from the person shows next stimulus and finally calculates the threshold [**11**]. This test was performed by two gratings and Landolt C stimuli for every person. Due to time-consuming tests, in this study, fatigue and loss of concentration were respectively 20 and 16 times higher in all three frequencies for gratings and Landolt C stimuli. The duration of display was 15 seconds for each stimulus. Measurement was performed at three frequencies of 1, 5, and 15 cycles of degree and under low mesopic light condition. Also, according to the standard of the test, as well as the resolution of the 15-inch computer screen test, the distance was 125 cm for grating stimulus at four directions (horizontal, vertical, diagonal two directions) and Landolt C at eight directions randomly. 

Color vision test: D15-Fransworth of color vision test was used to assess color vision. There was no time limit to perform these tests for people. Color vision test was performed monocularly and under high mesopic light condition and at 50 cms distance from persons. First, a correct arrangement of caps was shown to the person and after moving randomly all caps except for the first cap (guide cap), then the person was asked to consider color similarity caps and put them in order in the box. Test results were reported based on color confusion index (CCI). Color confusion index is calculated by dividing the sum of distances between color caps that the examiner arranges in Farnsworth D-15 test Instant standard sum of distances between correct arrangements. The minimum value of color confusion index is equal to 1 and the values higher than 1 indicate that color differentiation is worse [**12**,**13**].

Data was analyzed using version 22 SPSS software. For descriptive data, descriptive statistics indicators such as figures, frequency distribution tables, mean and standard deviation, were used. Independent t-test was used for evaluating the mean difference of contrast sensitivity and color vision between the two groups.

## Results 

All the participants in this study were males. The mean age was 31.49 ± 4.41 (20-50) years and the work experience was 6.13 ± 4.44 (1-27.17) years. In this study, 230 subjects were evaluated, among whom 140 (60.9%) were exposed to minerals and 90 (39.1%) were not exposed to minerals (administrative sector, security, warehouse and drivers). **Table 1** compares studied groups in terms of average age and work experience. As the table indicates, there were no significant differences between groups in terms of age and work experience (p˃0.05).

Contrast sensitivity test was performed for 277 eyes of the case group and 177 eyes of the control group. According to Freiburg contrast sensitivity test, there was a significant difference between the studied groups with Landolt C stimulus in all three frequencies of 1, 5, and 15 cycles per degree. With the grating stimulus, there was a significant difference between the two groups in frequencies of 1 and 15 cycles per degree but at a frequency of 5 cycles per degree, there was a border difference.

**Table 1:** Mean and standard deviation of RNFL thickness of diabetic patients undergoing conventional PRP (pre-PRP and post-PRP) in studies using OCT

	groups		
variable	case	control	P-value
Age (years)	31.18 ± 4.22	31.96 ± 4.65	0.07
work experience (years)	5.84 ± 3.79	6.58 ± 5.26	0.10

**Table 2** represents the comparison of the log contrast sensitivity between the two groups in all three frequencies with two stimuli.

**Table 2:** Comparison of log contrast sensitivity between the two groups

		groups		
Stimulus	Frequency (cycle/ degree)	case	control	P-value
Landolt C	1	1.71 ± 0.17	1.76 ± 0.18	0.009
	5	1.73 ± 0.18	1.78 ± 0.19	0.016
	15	1.69 ± 0.16	1.74 ± 0.17	0.003
Grating	1	2.28 ± 0.06	2.30 ± 0.00	<0.001
	5	2.26 ± 0.12	2.28 ± 0.07	0.051
	15	1.76 ± 0.36	1.84 ± 0.30	0.008

**Fig. 1:**
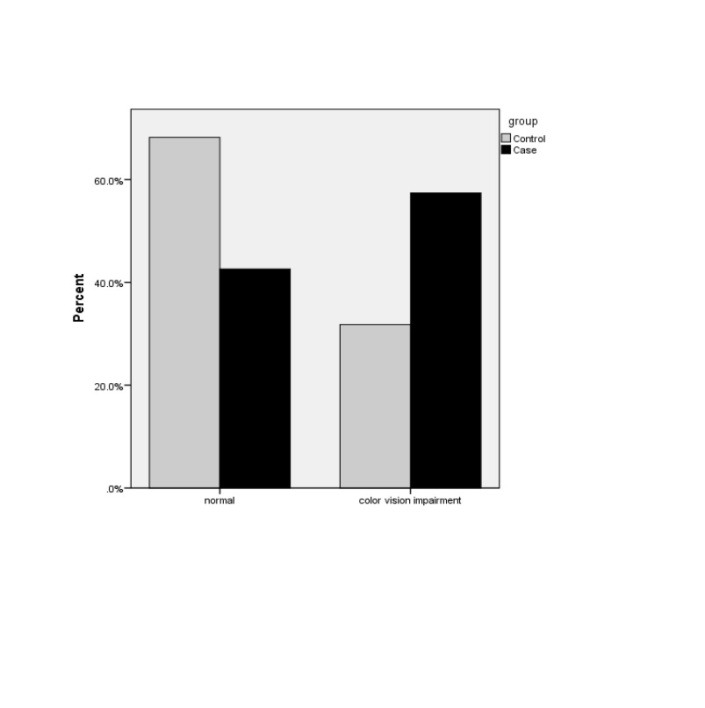
Status of color vision for the two groups

Color vision test was performed for 277 eyes of the case group and 177 eyes of the control group. According to Farnsworth D-15 test, the frequency percentage of color vision defect was present in control and case groups and were of 31.8% and 57.4% respectively. **Fig. 1** shows the frequency percentage color of vision impairment (CCI ˃1) in both groups.

A significant difference was observed between the color confusion indexes of the two groups (p<0.0001). The mean color confusion index was 1.16 ± 0.28 and 1.07 ± 0.14, respectively, in the case and control groups.

**Fig. 2:**
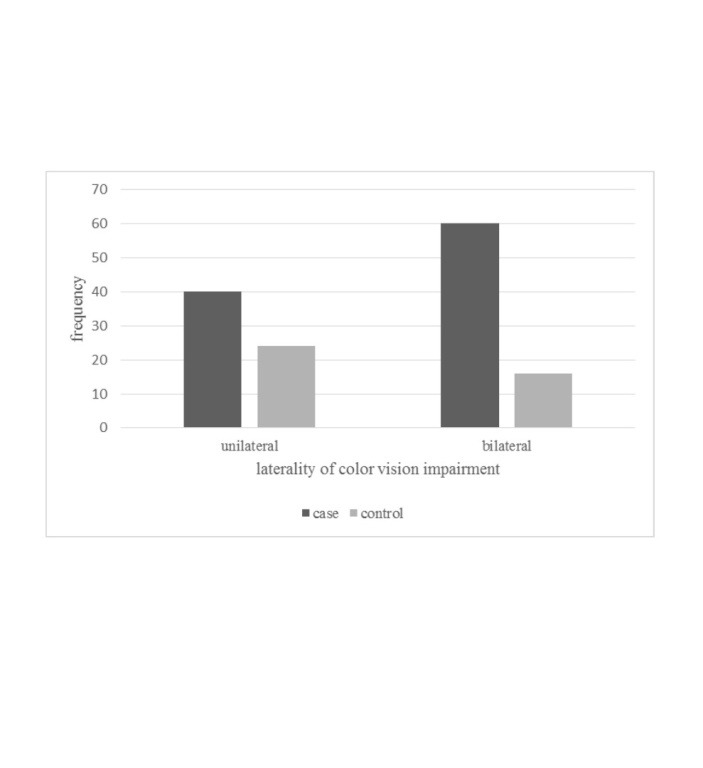
Laterality of color vision impairment in the two groups

**Fig. 2** shows the frequency of unilateral or bilateral color vision impairment in control and case groups.

## Discussion

Previously, most studies have focused on the contrast sensitivity study after occupational exposure has used grating stimulus. According to the duration of exposure and type of materials, which were often industrial compounds and heavy metals, different results have been reported.

Costa et al. studied the organic solvents effect on contrast sensitivity with sinusoidal grating stimulus in gas station workers with work experience of 9.6 ± 6.2 years and similar to the present study contrast sensitivity defects were observed at all frequencies [**7**]. Costa evaluated contrast sensitivity by sVEP following exposure to mercury (average exposure of 8.9 ± 4.1 years), the only impairment being seen in moderate spatial frequencies [**14**]. Also, Bowler et al. studied the contrast sensitivity by Vistch Contrast Sensitivity, which used a grating stimulus after the exposure to manganese in welding, the impairment being reported at low and moderate frequencies [**5**].

The results of this study showed that although visual acuity of workers who were in contact with minerals was more than 8/ 10 and the work experience 5.84 ± 3.79 years depending on the type of stimulus and the special frequency compared with the group that was not exposed to minerals, there was a significant difference in all three spatial frequencies of 1, 5, and 15 cycles per degree with Landolt C stimulus. Also, there was a significant difference at frequencies of 1 and 15 cycles per degree by grating stimulus and border disputes were found in the frequency of 5 cycles per degree between the two groups (**Table 2**).

In addition to contact with minerals in different parts, workers use chemical composition such as gasoline, Methyl Isobutyl Carbinol, Potassium ethyl xanthate, Potassium amyl xanthate, iron sulfate, copper sulfate and lime to purify lead and zinc in mineral processing.

In this study, all participants had a visual acuity of 8/ 10 and it evaluated one’s ability in high-contrast [**15**]. However, the group that had a higher exposure to minerals than the control group had lower contrast sensitivity in all frequencies. This indicated that the contrast sensitivity test was more comprehensive compared with visual acuity and could not rely only on visual acuity in the assessment of visual impairments.

Contrast sensitivity defect may indicate a chronic and presumably irreversible prolonged exposure and damage to the neuro-optic pathways [**16**]. In moderate frequencies, this defect may indicate changes in neuro-optic transmission that can be detected with visual acuity but if they are widespread [**2**]. The magnocellular system is responsible for processing low spatial frequencies, while high spatial frequencies are processed preferably by Parvocellular system. Since the high and low spatial frequencies are both affected by minerals, there are two possibilities for this to happen: 1. damage to the external retina before the separation of the two visual pathways or 2. entire visual pathway affected sporadically.

The quantitative assessment of color vision showed that there is color vision impairment in workers who were exposed to minerals compared with the control group.

Since studies have rarely been conducted about the exposure to minerals, the results of this study were fully consistent with the studies that used different tests to evaluate the color vision in the field of occupational exposure to various industrial materials and heavy metals, be they quantitative or qualitative.

Several studies investigated the effect of exposure to various materials such as industrial materials, chemicals and heavy metals on color vision by tests such as Lanthony D15-d, Farnsworth D-15, Ishihara, Cambridge Color and FM100, be they quantitative or qualitative, and all reported a color vision impairment in the exposed group. In all these studies, color vision impairment was yellow-blue color vision defect and there were yellow-blue and red-green color vision defects at the same time in some of them [**5**,**17**-**22**]. 

In this study, there was a significant difference between the amounts of CCI calculated from the two groups (0.0001 <P) and, similar to other studies in the field of occupational exposure, it could be stated that the exposure to minerals and related chemicals for the purification of inorganic minerals had an adverse impact on color vision and could lead to the acquired color vision impairment.

Regarding the acquired color vision impairment, this disorder can involve eyes unequally or monocularly, and it can be progressive or reversible depending on various factors [**23**]. In this study, color vision impairment was unilateral (n=40) and bilateral (n=60) with different CCIs for the two eyes in the exposure to minerals group.

Yellow-blue and red-green color differentiation respectively was observed by koniocellular and parvocellular pathways.

The determination of the colored status (red-green or yellow-blue color vision defect) - acquired color vision impairment is considered a sign of neural damage area. According to some studies, blue-yellow spectrum defect (indicating an acquired color vision impairment) shows involvement of external retinal layers, while green-red spectrum defect (indicating congenital color vision impairment) is caused by involvement of internal retinal layers or optic nerve [**3**,**6**,**22**]. 

Koushk mine environment contains a large amount of galena, which includes lead and sulfur. Lead is a heavy metal. According to a study conducted by Erie et al., lead can accumulate in retinal pigment epithelium, choroid and ciliary body. Lead-induced neurotoxicity is worrying, especially since even very low concentrations of lead could have harmful profound neurological effects. Exposure to low levels of lead cause a deterioration of scotopic vision and cylindrical and bipolar cells apoptosis. In rabbits, lead poisoning causes swelling of retinal pigment epithelium, leading to loss of photoreceptors. Visual impairment in humans, after lead induced systemic toxicity due to toxic effects of lead on the brain and optic nerve, is usually related to encephalopathy and optic neuropathy [**24**].

Besides lead, sulfur and organic gases can be found in the underground mining and mineral processing environment. Sulfur affects the anterior surface of the eye and acidifies the PH of the tear and symptoms such as burning and itching eyes [**25**], in VEP waves as well as in lens and retina changes [**26**].

Since the retina and its cells are involved in contrast sensitivity and color vision, their change will disrupt these two functions. As far as lead is concerned, saving the mine and the mine environment, which consists of sulfur and organic gases mostly, visual impairment (contrast sensitivity and color vision) followed by damage to retina layer is expected, and the results of this study proved it.

## Conclusion

Since different minerals have different environmental effects and there are different neurotoxic substances as well as exposure to gases, chronic occupational exposure to minerals and the material used to purify mineral ores may be emitted during extraction or may be absorbed through the respiratory system, eyes, skin and digestive system.

So, it is expected that with respect to a variety of different materials as well as their different properties such as the influence of the texture, intensity neurotoxic effects, volume density of materials, stability in the natural environment and other characteristics of these materials, different effects have existed on color vision and contrast sensitivity.

According to working conditions and necessity of health and safety, it may be possible to minimize damaging conditions by creating decent working conditions and secure protection and exact and sensitive examination. Color vision and contrast sensitivity tests are subclinical and used for the early diagnosis of neurodegenerative and visual disorders.

According to portability and ease of use, D15-Fransworth and Freiburg contrast sensitivity tests are considered for checkups and screening examination.

**Acknowledgements**

We are grateful to the noble workers and personnel of the Koushk mine, especially engineer Ahmadi and engineer Heydari.

**Financial Support**

This project was supported by Noor Research Center for Ophthalmic Epidemiology.

**Conflict of Interest**

No conflicting relationship exists for any author.
